# Janus kinase inhibitors are potential therapeutics for amyotrophic lateral sclerosis

**DOI:** 10.1186/s40035-023-00380-y

**Published:** 2023-10-12

**Authors:** Peter J. Richardson, Daniel P. Smith, Alex de Giorgio, Xenia Snetkov, Joshua Almond-Thynne, Sara Cronin, Richard J. Mead, Christopher J. McDermott, Pamela J. Shaw

**Affiliations:** 1 BenevolentAI, 4-8 Maple Street, London, W1T 5HD UK; 2 BenevolentAI, 15 MetroTech Centre, 8th FL, Brooklyn, NY 11201 USA; 3 Sheffield Institute for Translational Neuroscience, Faculty of Medicine, Dentistry and Health, University of Sheffield, Sheffield, UK; 4 NIHR Sheffield Biomedical Research Centre, University of Sheffield and Sheffield Teaching Hospitals NHS Foundation Trust, Sheffield, UK

**Keywords:** Janus kinase, STAT3, Amyotrophic lateral sclerosis

## Abstract

Amyotrophic lateral sclerosis (ALS) is a poorly treated multifactorial neurodegenerative disease associated with multiple cell types and subcellular organelles. As with other multifactorial diseases, it is likely that drugs will need to target multiple disease processes and cell types to be effective. We review here the role of Janus kinase (JAK)/Signal transducer and activator of transcription (STAT) signalling in ALS, confirm the association of this signalling with fundamental ALS disease processes using the BenevolentAI Knowledge Graph, and demonstrate that inhibitors of this pathway could reduce the ALS pathophysiology in neurons, glia, muscle fibres, and blood cells. Specifically, we suggest that inhibition of the JAK enzymes by approved inhibitors known as Jakinibs could reduce STAT3 activation and modify the progress of this disease. Analysis of the Jakinibs highlights baricitinib as a suitable candidate due to its ability to penetrate the central nervous system and exert beneficial effects on the immune system. Therefore, we recommend that this drug be tested in appropriately designed clinical trials for ALS.

## Introduction

Amyotrophic lateral sclerosis (ALS) is an archetypal multifactorial neurodegenerative disease whose primary cause in the majority of cases is still unknown. ALS is characterised by the loss of lower and upper motor neurons resulting in progressive failure of the neuromuscular system, with death usually occurring approximately 3–5 years after diagnosis due to the failure of the respiratory muscles [1]. A significant number of patients also show disturbed frontal and temporal lobe function [2] with some (approximately 5%) developing overt frontotemporal dementia. The disease process likely starts a significant period of time before symptoms first appear, suggesting that the functional changes identified in patients will include both disease processes and potentially beneficial compensatory adaptations.

ALS disease prevalence is approximately 9 per 100,000 persons [3], of which 5%–10% of individuals have familial ALS (fALS) defined by their family history. The most commonly associated genes in fALS are *C9ORF72, TARDBP, FUS* and *SOD1*. Recent genetic analyses have revealed numerous mutations in sporadic ALS cases, which reveal possible underlying genetic drivers of the disease [4]. Pathways implicated by such risk genes include apoptosis arising from mitochondrial dysfunction, autophagy with loss of protein homeostasis, inflammation (peripheral and central), impaired intracellular trafficking and excitotoxicity. These dysfunctional pathways operate in many cell types including the upper (corticospinal) and lower (spinal) motor neurons themselves, associated glial and Schwann cells, skeletal muscle fibres and their associated progenitors as well as cells of the immune/ inflammatory system. Considering the wide range of pathways and cell types affected, efficacious therapeutics for ALS may need to target pleiotropic regulators able to modulate a broad range of defective cellular processes, particularly as the originating causes are not fully understood.

## Repurposing drugs for ALS

Drug repurposing is the use of already approved drugs for new indications. Given the long and expensive drug development process, drug repurposing provides a means of accelerating therapeutic discovery at a lower financial risk. This is mainly because the toxicological and pharmacokinetic risks are known at the outset, and the molecular optimisation processes (i.e., preclinical drug discovery) have been completed. Importantly, for a rapidly progressing disease, repurposing offers the possibility of treating patients currently with the disease, an unrealistic process if starting from scratch, given the 10–15-year timescale to bring a new chemical entity to market. In addition, rare diseases such as ALS may not provide a realistic opportunity to recover the enormous costs of bringing a new drug to market ($2-3B) [6], shifting the risk-reward profile unfavourably for large pharmaceutical companies. This may explain why most of the current ALS clinical trials are investigating drugs from small pharmaceutical and biotechnology companies [4].

Only four drugs have been approved by the FDA for ALS: riluzole, edaravone, and the combination therapy AMX0035 and tofersen. Riluzole may act by reducing glutamate-mediated cytotoxicity in the motor cortex and spinal cord and has been shown to increase survival by as much as 19 months [5]. Edaravone is an antioxidant which may delay progression without affecting survival. AMX0035 alleviates mitochondrial and endoplasmic reticulum stress while tofersen is approved for treating patients with *SOD1* mutations. Neither edaravone nor AMX0035 (which was approved by the FDA based solely on Phase 2 data) has been approved in Europe. There is therefore a significant and well recognised need for new disease-modifying treatments capable of reducing the rate of progression and increasing survival of individuals afflicted by ALS.

Recently, computer-enhanced methods for repurposing approved drugs have been developed, particularly in response to the recent COVID-19 pandemic [7, 8]. These include AI approaches [9] and unbiased laboratory methods [10] to identify the protein–protein interaction networks operating in the disease state. Zeng et al. [11] compiled a knowledge graph (KG) integrating scientific literature and drug properties to identify approved drugs which could be used to treat COVID-19. In this context, the BenevolentAI KG was used in early 2020 to identify the Janus kinase (JAK) inhibitor baricitinib as a combined anti-inflammatory and anti-viral agent for the treatment of hospitalised patients with COVID-19 [12–14]. Subsequently, following the ACTT2 [15] and COV-BARRIER [16] clinical trials, this agent was approved for use in this patient population and has been used worldwide.

The BenevolentAI KG includes the contents of more than 85 diverse and independent structured biomedical databases [17, 18]. These structured data sources are further enhanced by natural language processing (NLP) pipelines, which integrate data from more than 35 million scientific papers. This KG was built to broadly cover complex disease biology across therapeutic areas and help scientists to develop data-driven hypotheses. Although the KG and associated machine learning models were predominantly built for predicting novel targets for disease, the technology can also support the repurposing of known treatments for alternative indications.

## Disease mechanisms and pathways of ALS

To conduct an unbiased literature-wide search of the disease mechanisms identified in ALS and their potential regulators, we used the BenevolentAI KG to identify 500 genes most specifically associated with ALS using a metric focused on the probability of the co-occurrence of two words within the literature. Since ALS causes skeletal muscle atrophy, we also identified 500 most associated genes with muscle atrophy. Pathway enrichment using these genes revealed four pathway groups as likely to be fundamental to ALS disease processes: cytokine-mediated inflammation, autophagy, apoptosis, and FOXO signalling pathways. Other mechanisms implicated to a lesser degree included TLR4-mediated inflammation and glutamate-mediated neurotransmission (Table 1).

**Table 1:** Twenty pathways most associated with the top 500 genes of ALS and muscular atrophy

Pathways	ALS		Muscular atrophy		Geneset
	Genes	adjP	Genes	adjP	
Signaling by interleukins	87	5.84E−33	79	5.59E−28	472
Interleukin-4 and interleukin-13 signaling	42	8.06E−29	43	1.90E−30	108
Autophagy	32	4.70E−13	28	1.29E−10	149
Selective autophagy	24	4.70E−13	20	6.80E−10	80
Interleukin-10 signaling	19	6.14E−13	13	3.75E−07	47
Macroautophagy	30	6.14E−13	26	3.40E−10	134
Programmed cell death	37	7.69E−13	35	6.37E−12	208
FOXO-mediated transcription	20	3.22E−11	27	2.02E−19	65
Cellular response to chemical stress	33	7.08E−11	34	4.85E−12	194
Apoptosis	30	1.11E−09	29	1.52E−09	179
Regulated necrosis	17	1.93E−09	14	4.14E−07	56
Purinergic signaling in leishmaniasis	12	5.19E−09	9	4.81E−06	26
Cell recruitment (proinflammatory)	12	5.19E−09	9	4.81E−06	26
PINK1-PRKN-mediated mitophagy	11	1.03E−08	10	1.01E−07	22
Mitophagy	12	2.17E−08	11	1.49E−07	29
Defective intrinsic pathway for apoptosis	11	5.08E−08	12	1.52E−09	25
TRIF(TICAM1)-mediated TLR4 signaling	21	5.08E−08	16	3.00E−05	108
MyD88-independent TLR4 cascade	21	5.08E−08	16	3.00E−05	108
Post NMDA receptor activation events	18	6.62E−08	8	0.036	80
Diseases of signaling by growth factor receptors and 2nd messengers	45	7.91E−08	61	2.85E−17	433

adjP, adjusted *P* value; Genes, number of mechanism-relevant genes among the ALS and muscular atrophy top 500 genes; Geneset, total number of genes associated with the pathway

**Fig. 1:**
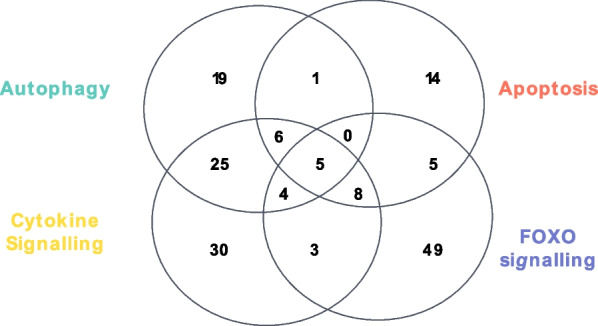
Genes common to ALS and atrophy mechanisms. Considering those ALS-associated genes which were identified as contributing to autophagy, apoptosis and cytokine signalling and the muscular atrophy genes of the FOXO signalling pathway revealed five ALS genes (*STAT3*, *HSP90AA1*, *FASLG*, *UBC* and *AKT1*) in common

However, of particular interest were the ALS-associated and muscle atrophy-associated genes which are common to these four processes. These were *STAT3* (encoding signal transducer and activator of transcription 3, STAT3)*, AKT1* (AKT serine/threonine kinase 1)*, HSP90AA1* (heat shock protein 90 alpha family class A member 1)*, UBC* (ubiquitin C) and *FASLG* (Fas ligand) (Fig. 1), indicating that STAT3 signalling is fundamental to the disease.

In addition to inflammation, autophagy, apoptosis and the FOXO pathways, JAK/STAT signalling is also heavily implicated in the characteristic pathophysiology of ALS, i.e., TDP43 protein aggregates, mitochondrial dysfunction, skeletal muscle denervation and excitotoxicity (Table 2). These disease processes are widely believed to be major contributors to ALS disease especially mitochondrial dysfunction, which results in deficits in ATP supply, protein aggregation with 95% of patients having TDP43 aggregates, and muscle denervation and atrophy which eventually causes death. The discovery that all these processes involve JAK/STAT signalling suggests that this pathway could be a therapeutic target.

**Table 2:** The fundamental ALS disease processes and associated pathways and mechanisms

Disease process	Pathways/mechanisms involved
Mitochondrial dysfunction	JAK/STAT pathway
	Pyruvate metabolism
	Mitochondrial quality control
	Electron Transport Chain
Protein aggregate formation	JAK/STAT pathway
	Protein localisation
	Autophagy
RNA metabolism dysfunction	Nuclear cytoplasmic transport
	RNA splicing
Neuroinflammation and glial toxicity	JAK/STAT pathway
	Astrocyte and microglial activation
	Type 1 IFN activation
	NK cell activation
Denervation and Muscle atrophy	JAK/STAT pathway
	Autophagy
	IL-6 signalling
	Activin A/SMAD signalling
	FOXO3 pathway
Excitotoxicity	JAK/STAT pathway
	Synaptic glutamate clearance
	Glutamate receptor activity
	Cytoplasmic Ca buffering
ER stress	JAK/STAT pathway
	IRE1α/PERK/ATF6

ATF6, transcription factor 6; SMAD, Mothers against decapentaplegic transcription factor; IRE1α, inositol-requiring protein 1 alpha; ER, endoplasmic reticulum

**Fig. 2:**
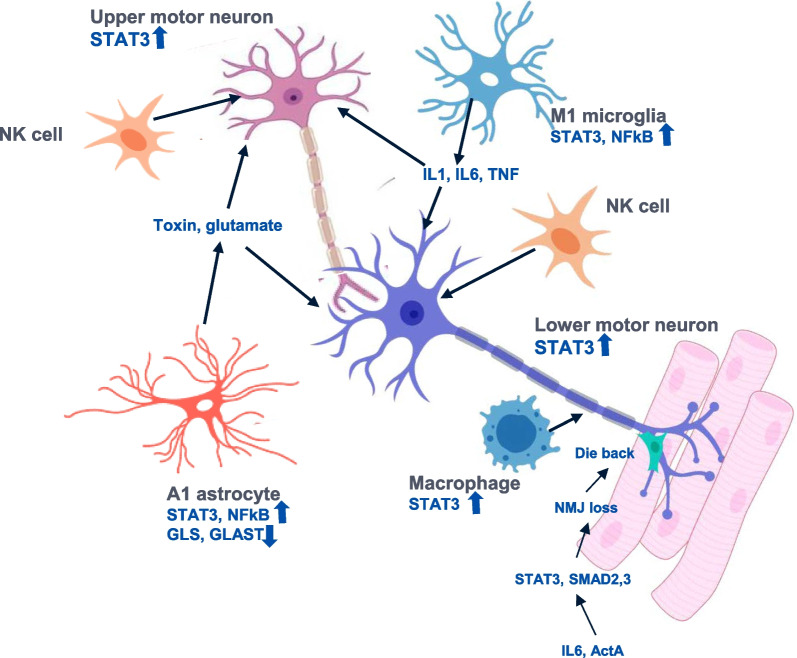
Non-cell-autonomous mechanisms of motor neuron loss in ALS. Non-cell-autonomous mechanisms within the motor cortex and spinal cord which contribute to motor neuron loss. The combined effects of microglial-derived cytokines, astrocyte-induced toxicity, NK cell cytotoxicity and macrophage phagocytosis of degenerating axons all need to be controlled. In addition, in the periphery, IL-6 and Activin A combine to induce disruption of the neuromuscular junction with consequent axonal die back due to loss of trophic support. Finally, chronically increased activated STAT3 contributes to motor neuron dysfunction through many of the processes summarised in Table 2. These mechanisms are reviewed in more detail below. ActA, activin A; SMAD2,3, mothers against decapentaplegic transcription factors 2 and 3; NMJ, neuromuscular junction; NK, natural killer; TNF, tumor necrosis factor; NFκB, NF-kappa-B; IL1/6, interleukin 1/6

In addition to the complexities inherent in modifying multiple disease processes, ALS treatment also requires that effective drugs have beneficial effects in a wide range of cell types where modulation of pleiotropic signalling pathways could have detrimental or even counter-productive effects. It is therefore important to assess the effect of drugs modifying JAK/STAT signalling in the cell types affected by the disease. The cell types and processes involved in ALS include glial cells, T cells (particularly NK cells), macrophages as well as the upper and lower motor neurons (Fig. 2).

In the following discussion the reader is referred to some recent excellent reviews for a comprehensive description of ALS [4, 19, 20], and we focus here on the potential role of STAT3 in the disease.

## STAT3 signalling in ALS

STAT transcription factors, primarily activated by JAKs, play a central role in transducing diverse immune signalling pathways. Regulation of STAT3 through phosphorylation, methylation and acetylation impacts various cellular functions, including gene transcription control, mitochondrial activity, calcium buffering, and apoptosis inhibition. The canonical STAT3 pathway mediates many of the actions of cytokines and is induced by JAK-mediated tyrosine phosphorylation (Y705), followed by STAT3pY705 dimerisation, nuclear import and the regulation of gene expression. This activation of the JAK/STAT pathway predominates in the post-mortem spinal cords of ALS patients where interleukin (IL)-6, IL-2, interferon (IFN) γ, IFNα and the unfolded protein response (UPR) pathways are all upregulated. Since JAK/STAT signalling is an integral part of these pathways, it is likely that JAK inhibitors will have a significant effect on their activity [see Fig. 1f of reference 21]. The non-canonical pathway is activated by serine/threonine kinase (e.g. extracellular signal related kinase [ERK], CDK5)-mediated phosphorylation of S727. The pY705/pS727 dual phosphorylation is required for full nuclear gene transcription activity [22]. The pS727 form performs distinct functions in the mitochondria as part of Complex 1 of the Electron Transport Chain (ETC) and is required for full ETC function while the cytoplasmic unphosphorylated form regulates microtubule stability [23].

### Canonical STAT3 signalling limits mitochondrial STAT3 (mitoSTAT3) signalling

**Fig. 3:**
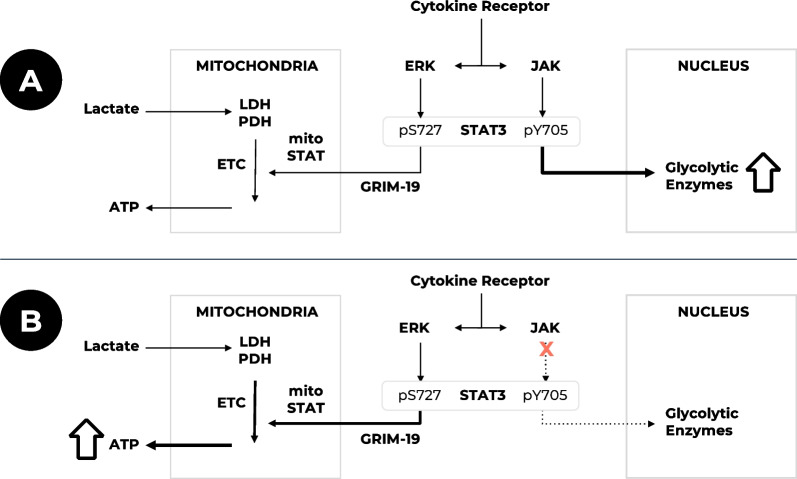
JAK inhibitors modify STAT3 signalling in motor neurons. **a** Under constant stimulation, STAT3pY705 accumulates in the nucleus, maintaining the expression of glycolytic enzymes but reducing the provision of mitoSTAT to the mitochondrial ETC, resulting in reduced oxidative phosphorylation and thus reduced ATP provision. **b** JAK inhibition reduces STAT3pY705, increases the provision of mitoSTAT3 to the mitochondrial ETC, thereby increasing oxidative phosphorylation and resulting in increased ATP generation. ERK, extracellular signal-related kinase; ETC, electron transport chain; GRIM-19, genes associated with retinoid–IFN-induced mortality-19; JAK, Janus kinase; LDH, lactate dehydrogenase; mitoSTAT, mitochondrial STAT3; PDH, pyruvate dehydrogenase; STAT3, Signal transducer and activator of transcription 3

The import of pS727 into mitochondria is mediated by the protein product of the *NDUFA13* gene known as Genes associated with Retinoid–IFN-induced Mortality-19 (GRIM-19) [24]. GRIM-19 overexpression reduces nuclear localisation of STAT3 [25] and nuclear STAT3-mediated gene expression [25–27], i.e., GRIM-19 inhibits the nuclear actions of STAT3 while enhancing its mitochondrial functions. This implies that STAT3 localisation may be dependent on the relative abundance of the pY705 and pS727 forms, with excessive pY705 generation potentially reducing the mitochondrial localisation of pS727 (Fig. 3). This has been suggested to occur in patients with gain-of-function mutations of *STAT3* who, surprisingly, show some immunodeficiencies. In these patients, the excessive dimerisation of STAT3 [28] and its consequent nuclear localisation will reduce mitoSTAT3, which may thereby cause the observed immune deficiencies due to compromised T cell function [29].

Unphosphorylated STAT3 is involved in the regulation of lysosomal function, and excessive nuclear localisation of STAT3 restricts this activity through lowering cytoplasmic STAT3 levels [30, 31]. The elevated plasma and CSF levels of pro-inflammatory cytokines in ALS [32, 33] are probably responsible for the high nuclear levels of STAT3 observed in post-mortem ALS microglia and astrocytes and in SOD1-G93A mouse motor neurons [34]. Both cytokines (e.g., IL-6) and reactive oxygen species induce high levels of nuclear STAT3 [35, 36] and are associated with a transient loss of mitoSTAT3. This cytokine-induced loss of mitoSTAT3 can be prevented by the JAK inhibitor ruxolitinib [36], which is consistent with the fact that the JAK-induced pY705 form limits the activity of mitoSTAT3 (Fig. 3). However, restoration of mitoSTAT3 after the cytokine or ROS action requires the mitochondrial membrane potential (MMP) to be maintained [24]. Since the MMP is compromised in ALS [37], the restoration of mitoSTAT3 will be deficient. A possible contributor to the defective mitochondrial function seen in ALS is therefore the loss of mitoSTAT3, due to excessive nuclear localisation of STATYp705 arising as a result of continuous cytokine signalling or ROS generation, and/or the reduction in the MMP.

Much of the evidence for a pivotal role of STAT3 in this disease is derived from animal models bearing causative mutations or from iPSC-derived motor neurons, with some post-mortem data from patients. Most of the data are therefore directly applicable to the more accessible lower motor neurons. However, many of the ALS disease processes listed in Table 2, including neuroinflammation [32, 38], lysosomal dysfunction with TDP43 protein aggregate formation [39] and consequent RNA metabolism dysfunction, mitochondrial dysfunction and endoplasmic reticulum dysfunction [40], have also been observed in upper motor neurons. We therefore suggest that much of these data are equally applicable to upper motor neurons which are hyperexcitable in ALS [41].

## Improving mitochondrial function

ALS-associated dysfunction of mitochondrial proteins has been identified in *SOD1, VCP, CHCHD10* and *TBK1* subtypes of ALS, indicating that mitochondrial dysfunction may be causative in ALS. Motor neuron mitochondria in patients and a range of models bearing ALS mutant genes are morphologically abnormal with fragments and aggregates throughout the axons [37]. The accumulation of these defective (but probably ROS-producing) mitochondria with abnormal cristae [42] is observed in patients and in those models with mutated or overexpressed *SOD1, C9ORF72, FUS* and *TDP43*. There are downstream consequences of this mitochondrial damage, including cGAS/STING-mediated inflammation, fragmentation, aberrant mitochondrial transport within neurons, reduced oxidative phosphorylation and ATP generation, reduced calcium buffering capacity, increased ROS generation and activation of the cellular apoptotic pathways. Since these mechanisms reinforce each other, therapeutic agents will need to inhibit multiple processes to effectively restore mitochondrial function. This could potentially be achieved through inhibition of the JAK/STAT signalling coupled with increased mitoSTAT activity as discussed below.

The mitochondrial defects in ALS are associated with the accumulation of protein aggregates and with increased inflammation. The latter may be partly a consequence of inflammaging (i.e., the gradual increase of inflammation seen with ageing [43]) in this age-associated disease, with a significantly higher degree of inflammation than in age-matched controls [32]. Increased levels of proinflammatory cytokines have been reported in ALS plasma [32] and CSF [33], indicating that these cytokines could stimulate upper and lower motor neurons as well as glial cells in both the motor cortex and spinal cord. Increased STAT3 activation has been shown in neurons and glia in post-mortem ALS samples [44], and in *SOD1* mutant mouse motor neurons [45]. Systemic cytokine/STAT3 signalling in ALS could therefore explain the mitochondrial dysfunction observed in many cell types including neurons, glia, muscle fibres and blood cells [46–49]. Similarly, canonical STAT3 activation and/or mitoSTAT3 depletion could contribute to poor glucose metabolism in motor neurons, poor mitochondrial quality control, reduced ETC activity and poor calcium buffering as outlined below.

### Restoring mitoSTAT3

**Fig. 4:**
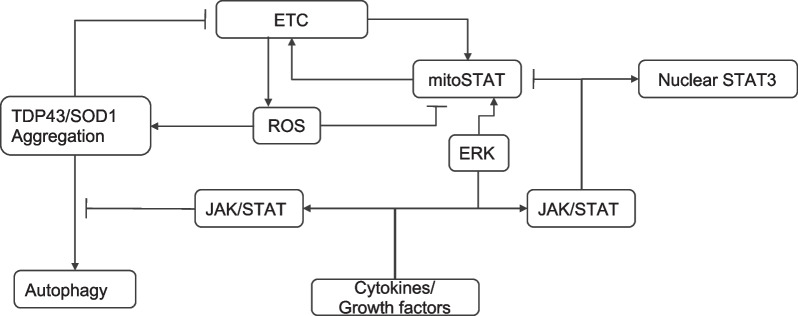
Self-perpetuating positive feedback loops contributing to mitochondrial deficiency in ALS. Two interacting and self-perpetuating feedback loops are envisaged: ROS-mediated protein aggregation which reduces the ETC resulting in further ROS, and ROS-induced mitoSTAT3 depletion which causes a reduction in the ETC thereby preventing mitoSTAT3 restoration. Note that impaired mitochondrial function leads to increased ROS

There are two potentially self-perpetuating disease mechanisms in ALS which cause mitochondrial damage. The first is ROS-induced protein (TDP43 and SOD1) aggregation with consequent mitochondrial damage and more ROS production [37, 50, 51]. The increased ROS production further induces protein aggregation, completing the feedback loop. Inhibition of JAK/STAT signalling would break this vicious cycle by promoting the autophagic clearance of the protein aggregates. The second is JAK/STAT-induced suppression of the ETC, exacerbated by the loss of mitoSTAT3 (Fig. 4). Since restoration of the mitoSTAT3 levels requires a functional ETC-driven MMP [24], the repeated depletion of mitoSTAT3 caused by cytokines would combine with the reduced MMP to result in ETC deficiency with downstream consequences on ATP generation and calcium handling. This second positive feedback loop would therefore be created by ROS-induced mitoSTAT3 depletion, which compromises the ETC and the MMP. This then reduces the ability of the mitochondria to recruit mitoSTAT3. JAK inhibition would restore mitoSTAT3 provision thereby restoring the activity of the ETC. Therefore, both of these feedback loops could be corrected by JAK inhibition, promoting increased provision of mitoSTAT3, increasing Complex 1 activity and thereby reducing ROS production and protein aggregation.

### Improving neuronal glucose metabolism

The high levels of STAT3pY705 in ALS tissues limit the generation of ATP through the Krebs cycle and oxidative phosphorylation by inhibiting the nuclear expression of ETC genes [52] and by increased expression of pyruvate dehydrogenase kinase 4 (PDK4) [53]. The latter inhibits activity of pyruvate dehydrogenase, thereby preventing the entry of glucose-derived acetylCoA to the Krebs cycle. This reduced glucose utilisation will reduce ATP supply in motor neurons, while in muscle mitochondria it could perhaps explain the relative insulin resistance seen in ALS patients [54]. JAK inhibition is therefore expected to improve the generation of ATP by the Krebs cycle and oxidative phosphorylation, thereby increasing the provision of ATP to the upper and lower motor neurons and muscle fibres.

### Restoring lactate oxidation by motor neurons

Glial-derived lactate is a major energy source for motor neurons [37]. However, PDK4 expression induced by high levels of STAT3pY705 would reduce the ability of neurons to utilise the lactate, resulting in increased beta oxidation and ROS production, despite the fact that the ETC is not fully functional. This is consistent with high STAT3pY705 activity causing increased aerobic glycolysis with reduced oxidative phosphorylation in cell lines [55]. The deficient ETC caused by loss of mitoSTAT3 (and reduced expression of the genes encoding ETC components) would also prevent utilisation of the glial-derived lactate. Accordingly, JAK/STAT inhibitors could restore ETC component expression and mitoSTAT levels and thereby restore oxidative phosphorylation, using both glucose and lactate as fuels. Consistent with this, inhibitors of nuclear STAT3 restore the MMP, the expression of mitophagy proteins and the calcium regulation in cardiac muscles [56]. Similarly, mitoSTAT3 activation has been shown to restore the MMP after neuronal injury [57] and inhibit ROS-mediated injury [58].

### Improving mitochondrial quality control

The accumulation of abnormal mitochondrial fragments is at least partly due to an imbalance in the fusion and fission pathways of mitochondrial homeostasis [37, 59] and to a failure of compensatory mitophagy. Interestingly, IL-6/gp130 signalling regulates mitochondrial quality control and morphology through both nuclear JAK/STAT3 (fusion) and ERK/mitoSTAT3 (fission) [60, 61]. As discussed above, IL-6 signalling also induces the transient reduction of mitoSTAT3 [36] and causes mitochondrial dysfunction [62], as can other pro-inflammatory cytokines such as tumor necrosis factor alpha (TNFα) [63]. Inhibition of mitoSTAT3 by the mitochondria-targeted STAT3 inhibitors mitocur-1 and mitocur-3 [64] also causes mitochondrial fragmentation [65, 66]. It is therefore conceivable that loss of mitoSTAT3 disturbs the balance between fusion and fission exerted by IL-6 and other gp130-associated cytokines. Since selective inhibition of nuclear STAT3 through JAK inhibition prevents this loss of mitoSTAT3 [36], it may also reduce mitochondrial fragmentation.

### Improving the ETC

The canonical STAT3 signalling reduces ETC activity through HIF1-alpha induction, which limits the expression of ETC components and induces PDK4 [67–70]. In addition, mitoSTAT3 plays a fundamental role in regulating ETC Complex 1 and has been shown to increase the activity of the whole ETC [55]. The functional defects in the ETC contribute to the reductions of MMP, ATP generation and calcium buffering, which are seen in neurons [46, 71], muscles [47, 72, 73] and even lymphocytes [49] of familial and sporadic ALS patients as well as in murine models [37]. This ETC deficiency also causes increased ROS production by mitochondria and excessive ROS have been reported in *SOD1* mutant cells, in post-mortem ALS tissues [74] and in fibroblasts from ALS patients [75]. JAK inhibitors are predicted to increase the ETC activity through increased expression of ETC components and through restoration of Complex 1 activity through increased mitoSTAT3.

### Restoring cytoplasmic calcium regulation

Intracellular calcium dysregulation has been strongly implicated in ALS [76–78]. This is almost certainly a consequence of a reduction in the mitochondrial/endoplasmic reticulum (ER) buffering capacity [4] as well as reduced ATP provision. This is exacerbated in motor neurons by the excessive calcium permeability of α-amino-3-hydroxy-5-methyl-4-isoxazolepropionic acid (AMPA) receptors and elevated synaptic glutamate levels (see Reducing excitotoxicity section below) which result in increased calcium in the nerve terminals [77]. JAK/STAT inhibition, through restoring the ETC, reducing synaptic glutamate and maintaining cortical GABAergic drive (see Reducing excitotoxicity section below), will improve the mitochondrial buffering capacity and so reduce the elevated cytoplasmic calcium levels in both upper and lower motor neurons.

## Clearing aggregated proteins by autophagy

One of the characteristic aspects of ALS is the accumulation of protein aggregates and stress granules in the affected motor neurons which, as mentioned above, cause mitochondrial dysfunction, with increased ROS production. The most commonly aggregated protein is TDP43, the product of the *TARDBP* gene. This protein is involved in RNA splicing and is usually found in the nucleus, although it also engages in shuttling between the nucleus and the cytoplasm. In approximately 95% of ALS cases, cytoplasmic ubiquitinated TDP43 aggregates are observed [79], mostly in the absence of *TARDBP* mutations. TDP43 aggregates are seen in both upper and lower motor neurons, as well as other apparently unaffected neurons [80], but are not seen with *SOD1* mutations, which instead reveal SOD1 aggregates in the mitochondria. Similar cytoplasmic FUS aggregates are seen in the presence of *FUS* mutations. In all three cases, it appears that these cytoplasmic aggregates have toxic properties, together with dysfunctions of RNA splicing and nucleocytoplasmic transport as added complications. These observations clearly suggest a failure of autophagy and proteostasis. Many mutated genes associated with ALS are linked to the proteasome and autophagy, including *C9ORF72, OPTN, VCP, SIGMAR1, PARK9, SQSTM1, CHMP2B, UBQLN2* and *FIG4*, indicating that dysfunctional proteostasis may also induce ALS.

The TDP43 aggregates are associated with nuclear TDP43 depletion and accordingly likely with the dysregulation of RNA metabolism affecting thousands of genes [81]. The cytoplasmic aggregates so formed may also sequester RNA transport components essential for appropriate export of RNA from the nucleus [82] and along the axon [83]. The stress granules may be causative in the generation of the toxic protein aggregates [84], so restoration of normal autophagic clearance of stress granules [85] would seem a necessary requirement in therapy.

### JAK/STAT inhibitors increase autophagy

In many ALS models, increasing the autophagy throughput has beneficial effects [86, 87]. The use of autophagy enhancers such as rapamycin and trehalose protects ALS neurons and astrocytes, clear TDP43 and FUS79 stress granules, increase the survival of mice with mutation of *SOD1* [88], TDP43 [89] or *C9ORF72* [90], as well as increasing muscle function of mice with *VCP* mutations [91]. In a wide range of cells, inhibition of JAK/STAT signalling increases autophagy [92–94]. In iPSC-derived motor neurons, cytoplasmic TDP43 aggregates can be cleared by improved autophagy through STAT3 inhibition [95]. Similarly, stimulating lysosomal exocytosis with the 1-phosphatidylinositol 3-phosphate 5-kinase inhibitor apilimod also reduced protein aggregates in iPSC-derived motor neurons [96]. This suggests that defects in proteasome and lysosome/autophagy pathways are two mechanisms operating in diseased neurons and that JAK/STAT inhibition may provide a route to restore function of the pathways.

Skeletal muscle fibres also show the accumulation of cytoplasmic aggregates, particularly in *SOD1* mutant models [97]. Interestingly, restricted expression of hSOD1 in mouse muscle causes an ALS-like disease [98]. In addition, some immune cells also show signs of cytoplasmic aggregates, which therefore indicate that the autophagy/proteasome defects are not motor-neuron-specific but are widespread, as would be expected if the germ line mutations are causative. Overall, inhibition of JAK/STAT signalling will increase autophagy, resulting in reduced protein aggregate build-up. It will also reduce ROS-induced aggregation and even potentially induce the removal of the ROS-producing mitochondrial fragments through improved mitophagy. It is also probable that clearance of cytoplasmic TDP43 aggregates will rectify some of the defects in RNA metabolism and transport.

## Reducing neuroinflammation and ALS glial toxicity

There is increasing evidence that neuroinflammation plays a key role in neurodegenerative diseases including ALS. Consistent with this, high levels of MCP-1, IL-15, IL-17 and TNFα are seen in the CSF of ALS patients [33] and many of the ALS-associated proteins are abundantly expressed in immune cells [79]. There is also evidence linking TDP43 to inflammatory nuclear factor NF-kappa-B (NFκB) pathways [99], as well as evidence showing that inflammation induces the cytoplasmic localisation of TDP43 [100], illustrating the inter-related nature of the disease processes of ALS.

### JAK/STAT signalling in ALS astrocytes

In astrocytes which are predominantly glycolytic, disruption of mitochondrial function caused by ALS-associated *SOD1* or *FUS* mutations results in increased ROS production and disrupted morphology [101]. In addition, it is reported that compromised mitochondrial activity with reduced lipid oxidation causes increased STAT3 signalling and astrocytic activation [102].

Some of the ALS astrocytes of the spinal cord are in a toxic A1-like state, and activated astrocytes have been reported in the motor cortex of ALS patients and mouse models [38]. The A1 astrocytes are characterised by secretion of proinflammatory and toxic factors [103], with an augmented immune response including elevated IL-6 and IL-8 expression [104]. These mouse ALS A1 and hIPSC-derived A1 astrocytes, but not the protective A2 astrocytes, are associated with high levels of JAK/STAT signalling [105]. The A1 astrocytes also show low expression of glutamate transporters and glutamine synthetase, an effect prevented by STAT3 deficiency [106]. This implies that reductions of synaptic glutamate clearance could play a major role in the excitotoxic damage to motor neurons in ALS (see Reducing excitotoxicity section below). This also suggests that inhibition of JAK/STAT signalling may reduce the activated A1 phenotype of these cells [23].

ALS astrocytes have been shown to be toxic to motor neurons [107] although the precise mechanism(s) have not been elucidated. One potential mechanism involves the astrocyte-induced reduction of MHC1 expression, resulting in astrocyte-mediated motor neuron death [108]. This lack of MHC1 expression may also make motor neurons susceptible to damage by invading natural killer (NK) cells. NK cells are highly upregulated in post-mortem motor cortex and spinal cord tissues of ALS cases [109, 110], consistent with an autoimmune component of the disease. The NK cells may invade the spinal cord in response to the upregulated inflammatory mediators IL-15 and MCP-1 (which signal through the JAK/STAT pathway [111]). In this context, depletion of NK cells in the *SOD1* and TDP43 murine models reduces the pace of motor neuron degeneration [109]. The JAK1/2 inhibitor ruxolitinib inhibited TLR4-mediated NK cell activation [38], and tofacitinib (a JAK1/2/3 inhibitor) protected ALS motor neurons from NK cell-mediated cytotoxicity in vitro and reduced NK cell numbers in mice [112].

Astrocytes expressing *SOD1* G93A also show abnormal responses to the alarmin HMGB1 which can be released by stressed motor neurons. These astrocytes fail to respond by increasing BDNF or GDNF, trophic factors which could help sustain the motor neurons [113]. Since HMGB1 signals through TLR4, this could perhaps be explained by the predicted low levels of mitoSTAT3 in ALS, which is required for some aspects of TLR4 inflammatory signalling [114].

### JAK/STAT signalling in ALS microglia

Microglia accumulate in degenerating regions of the central nervous system (CNS), and a neuronal ER stress response with associated microglial activation precedes neurodegeneration [115]. Activated microglia have been described in post-mortem cortex and spinal cord samples of ALS patients [116]. The proportion of toxic M1-type microglia increases at the onset of disease in the *SOD1* mouse model [117], which may accelerate disease progression [118]. This is consistent with the effect of masitinib, an inhibitor of the colony stimulating factor 1 receptor, which is predicted to reduce microglial-mediated inflammation and has been shown to delay the disease progression when given as an add-on to riluzole for over 48 weeks [119]. These M1 microglia can induce the polarisation of astrocytes into the toxic A1-like state through secretion of IL-1, C1q and TNF [120]. Post-mortem spinal cord microglia from ALS cases show a high degree of STAT3 activation [44], and it has also been shown recently that STAT3pY705 inhibition reduces the microglial-mediated inflammation in the spinal cord [121].

### JAK/STAT signalling in the glial type 1 IFN response

Neuroinflammation may also be triggered by release of mitochondrial DNA from glial cells due to SOD1/TDP43-induced damage, resulting in activation of the inflammatory cGAS-STING pathway [122, 123]. Activation of this pathway is apparent in spinal cord samples from ALS patients and in the motor cortex of animal models [51]. The resultant type 1 IFN cytokines signal through JAK/STAT, suggesting that JAK inhibitors could reduce the proinflammatory effects of this pathway. Indeed, inhibition of this pathway reduces disease in ALS models [51]. In *C9ORF72* mutant models, the aberrant RNA metabolism, perhaps secondary to TDP43 malfunction, also causes inflammation through activation of the type 1 IFN response with consequent activation of JAK/STAT signalling. Type 1 IFN signalling induced by transfection of double-strand RNA (thus mimicking the effect of the repeat mutation in the *C9ORF72* gene) in human nerve cells was inhibited by JAKinibs [124]. Taken together, these data suggest that glial inflammatory damage and type 1 IFN-mediated damage to motor neurons would be reduced by inhibition of JAK/STAT signalling.

The projection of motor neurons into the periphery renders them susceptible to the action of peripheral immune system components including macrophages. In post-mortem tissues, macrophages are associated with the axons of motor neurons, and *SOD1-*mutant macrophages are associated with faster progression and reduced survival in mice [125]. In ALS patients, the monocytes adopt a pro-inflammatory phenotype with increased expression of IL-1β, IL-8 and SOCS3 [126], the latter suggesting significant JAK/STAT signalling. Interestingly, reducing peripheral macrophage activation also reduces central microglial activation, suggesting that modification of peripheral macrophages could reduce some of the central neuroinflammation [127]. IL-6 has been shown to increase the anti-inflammatory (M2) properties of human and mouse macrophages at least partly through ERK signalling, perhaps mediated by STAT3pS727 [128]. This is consistent with both ERK [129] and STAT3 signalling being the major regulators of macrophage polarisation. JAK inhibitors do, however, have anti-inflammatory effects on M1-like macrophages and anti-fibrotic effects on M2-like macrophages, positioning them as generalised inhibitors of macrophage function [130].

Finally, STAT3 also promotes the expression of NACHT, LRR and PYD domain-containing protein 3 (NLRP3) and so the generation of IL-1β, one of the pro-inflammatory cytokines highly expressed in ALS serum [32] and strongly implicated in neuroinflammation [131]. In summary, the JAK/STAT signalling is pivotal to ALS-associated neuroinflammation and inhibitors will significantly reduce the inflammatory response to the ALS disease processes.

## Reducing muscle denervation and atrophy

It has long been suggested that the failure of the neuromuscular junction (NMJ) is one of the early processes in ALS, with a probable consequent loss of trophic support between nerve and muscle, resulting in nerve “die back” and muscle atrophy. Since an ALS-like syndrome can be induced by muscle-specific expression of hSOD1, such aggregates may contribute to the denervation of the muscle, perhaps through loss of proteins such as rapsyn required for the maintenance of the post-synaptic component of NMJ [98]. The resultant denervation may then result in motor neuronal injury and progression of the disease in the spinal cord.

Other conditions in which the NMJ fails include sarcopenia and cancer cachexia where this failure precedes muscle atrophy [132]. Since the establishment of a functional NMJ requires the elimination of less active synapses on the muscle fibre, the denervation may induce dieback of synapses and axons, which is mediated by similar mechanisms as synaptic pruning. Such synaptic pruning in the CNS has recently been shown to be mediated by JAK2/STAT1 signalling in the corpus callosum [133], suggesting that increased cytokine/gp130 signalling (e.g. IL-6) could be responsible for the denervation seen in ALS.

Increased IL-6 activity is reported in ALS muscles, where increased synthesis of this myokine in fibro-adipogenic progenitors results in increased IL-6 production and STAT3 activation. Inhibition of IL-6/STAT3 prevents atrophy in the *SOD1* transgenic mice [134, 135] and in cancer cachexia models [136, 137]. Sartori et al. recently showed that in models of cancer-induced cachectic muscle wasting, the NMJ disassembly occurs before atrophy and is a consequence of STAT3-induced noggin expression [132]. The resultant inhibition of transforming growth factor beta family-mediated Mothers against decapentaplegic homolog (SMAD)1/5/8 signalling causes NMJ failure. This effect is synergistic with activin A- or myostatin-stimulated SMAD2/3 which also inhibits SMAD1/5/8 signalling, while increased SMAD1/5/8 signalling prevents the denervation. Similarly, Ma et al. [138] showed that JAK/STAT3 signalling mediates both IFN- and TNF-induced cachectic muscle wasting. Finally, the atrogenes responsible for muscle atrophy are induced by a combination of STAT3 and FOXO3 [139, 140]. It is therefore clear that STAT3 activation, mainly due to an increase in the myokine IL-6, can promote NMJ disassembly, which in turn further increases IL-6/STAT3 signalling [141], creating a positive feedback loop, resulting in muscular atrophy. In addition, this or related cytokines may mediate both the synapse elimination and the axonal dieback via JAK2/STAT1 [133].

Since respiratory muscle failure is a major cause of ALS-associated mortality, it is important to consider the effect of the JAK/STAT pathway on respiratory muscles. Mechanical ventilation of the lungs causes significant diaphragm dysfunction characterised by atrophy and a reduction in force which can result in ventilator dependency [142]. The mechanisms implicated are similar to those seen in ALS, i.e., apoptosis, autophagy, ROS and ROS-induced mitochondrial dysfunction, coupled with aberrant calcium handling and resultant calpain activation [143]. In both human cases and rodent models, mechanical ventilation is associated with high levels of STAT3 activation which induce mitochondrial dysfunction and ROS generation, both of which are suppressed by JAK inhibition [144]. The JAK inhibitor tofacitinib is currently being tested in a clinical trial of ventilator-induced diaphragm atrophy [145] (NCT03681275).

### STAT3 stimulation of axon regrowth

One consequence of ALS is the progressive loss of motor axons which appear to be unable to repair, regrow or reform appropriate connections with the muscle fibres. Many transcription and other factors have been implicated in axonal growth and regeneration including STAT3 [146]. MitoSTAT3 has been shown to significantly potentiate axonal regrowth in injured CNS neurons, together with the action of nuclear STAT3 [147]. Accordingly, under conditions of excessive ROS and cytokine action as seen in ALS, the predominance of nuclear STAT3pY705 may preclude the generation of adequate mitoSTAT3, thereby preventing appropriate axonal repair. Axonal repair may also be inhibited by the depletion of cytoplasmic unphosphorylated STAT3 under conditions of chronic cytokine and ROS stimulation, since it is required for microtubule stabilization [148]. This could be one of the reasons that chronic secondary inflammation after spinal cord injury prevents appropriate axonal regrowth (see for example Pang et al. [149]). Consistent with this, the JAK1/2 inhibitor ruxolitinib has been shown to improve motor recovery after spinal cord injury in mice [150], perhaps by normalising the balance between mitochondrial, cytoplasmic and nuclear actions of STAT3.

## Reducing excitotoxicity

The reduced ability of ALS A1-like astrocytes to clear synaptic glutamate [106] resulting in increased stimulation of excitatory glutamate receptor probably exacerbates the failure of the mitochondrial/ER mechanism to buffer cytoplasmic calcium in motor neurons. The combination of increased excitation and reduced calcium buffering will then contribute to the decline of these neurons. It has been suggested that motor neurons, due to their large size and plethora of excitatory calcium-permeable AMPA receptors, are particularly susceptible to excitotoxicity. Supporting evidence comes from the partial efficacy of riluzole which may reduce synaptic glutamate release by inactivation of Na^+^ channels [151], and from the detection of cortical hyperexcitability in ALS patients [4]. Calcium-permeable AMPA receptors are highly expressed in motor neurons [78, 152], putting extra strain on the calcium buffering capacity of these cells. Indeed, inhibiting the Q/R editing of the GluA2 subunit of the AMPA receptor through adenosine deaminase ADAR2 deficiency induces an ALS-like phenotype, reinforcing the idea that calcium mishandling contributes to this disease [153]. In addition, it appears that the CaV1.3 voltage-sensitive Ca^2+^ channel and unedited AMPAR GluA2 subunits are more highly expressed in ALS-sensitive motor neurons than in the more resistant oculomotor neurons [154]. Inhibiting JAK/STAT signalling will increase synaptic glutamate clearance by astrocytes [106], reduce cytokine-induced cytoplasmic calcium [155], and increase the calcium-buffering capacity (through restoration of the mitochondrial ETC), thereby reducing the excitotoxic stress on motor neurons.

Hyperexcitability of cortical neurons in ALS has been widely described as an aspect of the ALS disease, with a reduction in threshold potential resulting in increased excitability and reduced inhibition [156]. This hyperexcitability is associated with reduced GABA and increased glutamate/glutamine levels in the motor cortices of ALS patients [157]. Interestingly, in epilepsy models, inhibition of JAK/STAT signalling prevents the loss of GABAergic neurons [158], while in brain injury models, elevated STAT3 inhibits the expression of GABA receptor subunits [159] and STAT3 has been implicated in mediating the development of epilepsy-associated hyperexcitability [160]. The hyperexcitability may also be a consequence of JAK2-mediated inhibition of the Na^+^/K^+^-ATPase [161], which is the major driver of the membrane potential, suggesting that under conditions of high cytokine action (when JAK2 is active) the membrane of the motor neurons may become partially depolarised and so hyper-responsive to depolarising stimuli. The alpha3 subunit of the Na^+^/K^+^-ATPase is heavily implicated in ALS disease progression, particularly in the SOD1 models, since SOD1 aggregates bind this subunit [162], and prevention of such binding increases the Na^+^/K^+^-ATPase activity and extends survival presumably through reduced excitability [162]. These observations suggest that JAK/STAT inhibitors could reduce the hyperexcitability of upper motor neurons through maintenance of the cortical GABAergic inhibition and the polarisation of neuronal plasma membranes. However, increasing motor neuron excitability with AMPA receptor agonists can also extend survival [163], so it is not clear what role excitability plays in the disease [164]. It is tempting to speculate that these contradictory data may be a consequence of different stages of disease with hyperexcitability in early stages and a compensatory reduced excitability in later stages.

## Reducing ER stress

ER stress and UPR are activated by many ALS-related proteins, including SOD1, C9ORF72, FUS and TDP43, suggesting that this pathway may be a source of potential therapeutic targets. All 3 major pathways of the UPR interact with JAK/STAT signalling. Inhibition of JAK/STAT signalling directly inhibits the serine/threonine-protein kinase/endoribonuclease IRE1a and eukaryotic translation initiation factor 2-alpha kinase 3 (EIF2AK3 or PERK) arms of the UPR through blockade of type 1 IFN receptors [165] and PERK [166], respectively, while also reducing activating transcription factor 6 expression in the third arm [167]. Improvements in mitoSTAT3 provision to the mitochondria through JAK/STAT inhibition could also reduce ER stress [168].

In addition, the generation of misfolded dipeptide repeat proteins through repeat-associated non-AUG (RAN) translation of the RNA hexanucleotide repeat expansion in the *C9ORF72* mutation results in activation of ER stress and the integrated stress response. The widely used antidiabetic drug metformin has recently been shown to be effective in a model of *C9ORF72*-ALS [90] through inhibition of the ER stress-related IFN-induced, double-stranded RNA-activated protein kinase (PKR). However, the integrated stress response is also activated through PERK which signals through JAK/STAT, suggesting that a combination of a JAK inhibitor with metformin would be most effective in patients with dipeptide repeat and other misfolded proteins such as TDP43. The combination of improved protein aggregate clearance and inhibition of the downstream consequences of the UPR by JAK/STAT inhibitors will significantly reduce the effects of the ER stress response in motor neurons.

## JAK inhibitors

JAK inhibitors have been approved for the treatment of resistant rheumatoid arthritis (tofacitinib, baricitinib, filgotinib, upadacitinib) and myelofibrosis (ruxolitinib, fedratinib) [169]. Due to the involvement of upper motor neurons in ALS, it would be beneficial for the selected JAK inhibitors to be at least partially CNS-penetrant. In this context, the relatively low plasma-protein-binding of tofacitinib and baricitinib (approximately 50%) makes them good candidates, but tofacitinib has very low brain preparation (FDA Pharmacology Review). In contrast, baricitinib at therapeutic doses crosses the barrier in non-human primates (CSF concentration approximately 20% of total plasma concentration) [170], despite being a substrate of the CNS efflux pumps P glycoprotein (PGP) and a breast cancer resistance protein. Interestingly, progressive multiple atrophy is a form of motor neuron disease largely restricted to lower motor neurons which are exposed outside the blood–brain barrier, suggesting that high brain penetrance may not be required for this form of the disease. Other properties required for a JAK inhibitor treatment of ALS include inhibition of IL-6/gp130 (i.e. JAK1/2/Tyk2) and IFNα and β (JAK1/TYK2) signalling. JAK3 inhibition may also be beneficial through a reduction in NK cell and other pro-inflammatory cascades.

Although it would be better to use a fully brain-penetrant JAK inhibitor to reduce the dose required, there are no approved inhibitors of JAK1 and JAK2 with better brain access than baricitinib to our knowledge [171].

The side-effect profiles of the JAK inhibitors are variable. For tofacitinib and baricitinib, opportunistic and herpes zoster infections are the major adverse events in the context of prolonged dosing.

Long-term treatment (months to years) with JAK inhibitors is associated with side effects in a small number of patients, including venous thromboembolisms and Herpes infections [172]. In the clinical trials assessed by the EMA for the registration of baricitinib which included 4214 patient years, the most significant side-effect was a small increase of upper respiratory tract infections (similar to that observed with methotrexate). The incidence of Herpes zoster infections was low (3.2 per 100 patient-years), and similar to placebo [173].

However, in a 4-year study comparing tofacitinib and anti-TNF in patients aged over 50 years with rheumatoid arthritis in the presence of methotrexate, the JAK inhibitor showed an association with increased risks of cardiovascular issues (1.33-fold), cancers (1.48 fold) and herpes zoster reactivation (threefold) [174]. As a result, tofacitinib, baricitinib and upadicitinib have black box warnings for the risk of thrombosis and cancer [175]. In an analysis of 9 clinical trials in which some patients were dosed with baricitinib for up to 9 years, there were no significant differences for baricitinib versus placebo in the incidence of death, malignancy, serious infection, or major adverse cardiovascular events. The incidence per 100 patient years was 0.5 for venous thromboembolism, 0.3 for deep vein thrombosis, 0.2 for pulmonary embolism, and 1.0 for cancer [176]. Baricitinib has also been taken for brief periods by over one million seriously ill hospitalised patients with COVID-19. In meta-analyses, few, if any, drug-induced serious adverse events were reported in this patient population; in contrast, the drug showed significant mortality benefits even when added to other treatments such as steroids and tocilizumab [177]. In addition, the incidence of secondary infections with COVID-19 was reduced by 50% by baricitinib in the ACTT-2 clinical trial [178].

Baricitinib is approved in 2 mg and 4 mg oral formulations, has a half-life of 8–9 h and reaches a plasma Cmax (4 mg dose) of 112–135 nM, with 50% plasma protein bound, and equal plasma/blood equilibration implying free access to the inside of cells. The IC50 at JAK1/2 is 5.8 nM, Tyk2 53 nM and > 500 nM at JAK3. When dosing 4 mg qd the trough concentration is predicted to be 15 nM after a single dose with perhaps 2–3 nM in CSF. The effect of baricitinib on spinal cord inflammation in patients with ALS or Alzheimer’s disease is currently the subject of a clinical trial also examining the drug exposure in the CNS (NCT05189106).

## Conclusions

JAK/STAT3 signalling is central to many of the disease processes associated with ALS. JAK inhibitors are therefore predicted to have widespread beneficial effects in ALS, correcting many pathophysiological processes including: mitochondrial dysfunction (abnormal morphology, reduced MMP, poor calcium buffering, reduced ATP generation), autophagy (thereby correcting protein aggregate accumulation and dysfunctional RNA metabolism), and neuroinflammation mediated by astrocytes, microglia and NK T cells, while simultaneously reducing the risk of excitotoxicity, ER stress and cytoplasmic calcium overload. Since it appears that motor neuron damage may result from both direct effects on the nerve (e.g. mediated by glial cells) and muscle atrophy-induced denervation, it is important that JAK inhibitors are predicted to protect both the neuron and the muscle through multiple mechanisms, including those with self-perpetuating positive feedback mechanisms (NMJ denervation and mitochondrial dysfunction). There are FDA- and EMA-approved JAK inhibitors which have significant, but tolerable side effects, which are likely to have a positive risk–benefit ratio given the progressive and lethal nature of ALS. Finally, this approach to identifying inhibitors capable of simultaneously inhibiting the multiple disease mechanisms operating in ALS could become a model for identifying therapeutics in other multi-factorial diseases, including other neurodegenerative diseases.

## Data Availability

Not applicable.

## Abbreviations

ALS: Amyotrophic lateral sclerosis
AMPA: α-Amino-3-hydroxy-5-methyl-4-isoxazolepropionic acid
ERK: Extracellular signal related kinase
ETC: Electron transport chain
GRIM-19: Genes associated with retinoid–IFN-induced mortality-19
IFN: Interferon
JAK: Janus kinase
KG: Knowledge graph
mitoSTAT: Mitochondrial STAT3
MMP: Mitochondrial membrane potential
PDK4: Pyruvate dehydrogenase kinase 4
SMAD: Mothers against decapentaplegic transcription factor
STAT3: Signal transducer and activator of transcription
UPR: Unfolded protein response
